# Patient and treatment characteristics associated with patient activation in patients undergoing hemodialysis: a cross-sectional study

**DOI:** 10.1186/s12882-018-0917-2

**Published:** 2018-06-01

**Authors:** Liesbet Van Bulck, Kathleen Claes, Katrien Dierickx, Annelies Hellemans, Sofie Jamar, Sven Smets, Gijs Van Pottelbergh

**Affiliations:** 1 KU Leuven, Department of Public Health and Primary Care, Academic Centre for Nursing and Midwifery, Kapucijnenvoer 35 (box 7001), B-3000 Leuven, Belgium; 2 University Hospitals Leuven, Department of Nephrology and Renal Transplantation, Herestraat 49, B-3000 Leuven, Belgium; 3 KU Leuven, Department of Microbiology and Immunology, Laboratory of Nephrology, Minderbroederstraat 10 (box 1030), B-3000 Leuven, Belgium; 4 Imeldaziekenhuis, Nephrology, Imeldalaan 9, B-2820 Bonheiden, Belgium; 5 Sint-Trudo Ziekenhuis, Nephrology, Diestersteenweg 100, B-3800 Sint-Truiden, Belgium; 6 KU Leuven, Department of Public Health and Primary Care, Academic Centre for General Practice, Kapucijnenvoer 33 (box 7001), B-3000 Leuven, Belgium

**Keywords:** End-stage renal disease, Hemodialysis, Patient activation, Personalized interventions

## Abstract

**Background:**

Patient activation is associated with better outcomes and lower costs. Although the concept is widely investigated, little attention was given to patient activation and its predictors in patients undergoing hemodialysis. Hence, we aimed to investigate the level of patient activation and aimed to determine patient- and treatment-related predictors of activation in patients undergoing hemodialysis.

**Methods:**

This cross-sectional observational study recruited patients undergoing hemodialysis in three Flemish hospitals. Participants were questioned about patient characteristics (i.e., age, sex, education, employment, children, social support, leisure-time, living condition, and care at home), treatment- and health-related characteristics (i.e., hospital, time since first dialysis, transplantation, self-reported health (EQ-VAS) and depressive symptoms (PHQ-2)), and patient activation (PAM-13). Univariate and multiple linear regression analyses with dummy variables were conducted to investigate the associations between the independent variables and patient activation.

**Results:**

The average patient activation-score was 51. Of 192 patients, 44% patients did not believe they had an important role regarding their health. Multiple linear regression showed that older patients, who reported being in bad health, treated in a particular hospital, without leisure-time activities, and living in a residential care home, had lower patient activation. These variables explained 31% of the variance in patient activation. Based on literature, we found that activation of patients on hemodialysis is low, compared to that of other chronic patient groups.

**Conclusion:**

It could be useful to implement patient activation monitoring, since the level of activation is low in patients undergoing hemodialysis. Older patients, who reported being in bad health, treated in a particular hospital, without leisure-time activities, living in a residential care home, are at higher risk for lower activation.

**Electronic supplementary material:**

The online version of this article (10.1186/s12882-018-0917-2) contains supplementary material, which is available to authorized users.

## Background

Chronic kidney disease (CKD) is defined as structural or functional abnormalities of the kidneys, present for more than three months [1]. When renal function further deteriorates, patients develop end-stage renal disease (ESRD) with need for renal replacement therapy, i.e., hemodialysis, peritoneal dialysis, or renal transplantation [1].

The number of patients in need for a renal replacement therapy is increasing rapidly. In the United States in 2013, the prevalent dialysis population consisted of 466,607 patients [2]. This population has increased by 63.2% since 2000. On the other hand, dialysis treatments are very expensive. Hence, along with the increasing population, the cost of providing dialysis and transplantation continue to escalate [2, 3].

One way to influence health-care costs on the long-term could be to focus on patient activation [4]. Driven by the person-centered approach, patient activation specifies the level of patients’ involvement with their health care and refers to the extent to which they have the knowledge, belief, motivation, confidence, and skills to manage a chronic disease [5]. There is a growing body of literature indicating that activated patients make more effective use of healthcare services, have better self-management behaviors [6], better patient outcomes, better care experiences [7], and lower health-care costs [4] in chronic patients. Hence, to enhance patient outcomes at lower costs, the level of activation should be optimized.

In previous research, patients with end stage renal disease (stage 5), both with and without dialysis, had the lowest patient activation scores (average: 58) of all chronic kidney patients [8]. Due to the intensive dialysis treatment and proximity of healthcare workers, the patient activation of patients undergoing hemodialysis might be even lower. However, an assessment of the level of activation in a particular relevant growing population of expensive patients undergoing hemodialysis is lacking.

The level of activation can be improved using tailored coaching [9]. In order to be able to identify patients at high risk of low activation and in the context of the development of tailored interventions, an understanding of all patient- and treatment related factors associated with patient activation is needed [10]. Based on the Society to Cells Resilience framework [11], Gleason et al. [12] have shown that patient activation in an older adult population with functional difficulties was related to age, family support, difficulties with activities of daily living, depressive symptoms, self-reported health, and living situation, among other factors. It is still unclear whether these factors are also predictive for patient activation in patients undergoing hemodialysis. In addition, treatment-related factors, specifically for dialysis, have not yet been associated with patient activation.

Therefore, the present study was guided by two objectives. First, we determined the level of activation of patients undergoing hemodialysis. It was hypothesized that the average patient activation score would be lower than 58. In order to be able to interpret this score, an additional aim was to compare the level of activation of patients undergoing hemodialysis with the level of activation of other chronic populations. Second, we aimed to investigate the patient- and treatment-related characteristics associated with activation in patients with hemodialysis. It was hypothesized that better self-reported health, higher education level, and good social support would be associated with higher PAM. In addition, we hypothesized that higher age, no job, no hobbies, use of multiple home care services, and depressive symptoms would be associated with lower patient activation.

## Methods

### Design and study population

In this quantitative, observational, cross-sectional, and questionnaire-based study, convenience sampling was used. Participants were recruited in three Flemish dialysis units based on following criteria: (1) diagnosis of ESRD; (2) older than 18 years; (3) Dutch-speaking; (4) cognitively able to understand difficult concepts (at examiner’s discretion: first question contained the word ‘responsibility’ and the examination stopped when the participant did not understand that word correctly); (5) dialysis treatment longer than three months. Systematically, all hemodialysis patients that were treated in the hospital on the day of the investigation and that were eligible, were asked to participate.

To determine the sample size, the number of variables was examined. In order to achieve sufficient statistical power, 50 patients and an additional 8 patients for each variable had to be enrolled [13]. Because of the 15 variables in this study, the sample should thus consist of at least 170 patients.

### Data collection and ethics

Data were collected in February and March 2016. The questionnaires were completed independently or together with the interviewer. The same interviewer (LVB) was available on request for all patients. Self-reported questionnaires were used to measure all variables. Hence, data were gathered from the patient’s perspective [see Additional file 1].

Approval for the study was given by the Independent Commission for Medical Ethics of the UZ/KU Leuven, Medical Ethics Committee of hospital Imelda, and Medical Ethics Committee of hospital Sint-Trudo, all located in Belgium. Procedures were in accordance with the declaration of Helsinki [14]. Oral and written information about purpose, duration, and risks of study participation was given to patients before they were asked to participate. All participants provided written informed consent.

### Outcome measure

The primary outcome was patient activation, which was measured by ‘Patient Activation Measure-13’ (PAM-13, Dutch version) [15]. This instrument is a non-disease specific scale that shows involvement of patients in their health. This 5-item Guttman scale has following possible answers: ‘disagree strongly’ (1), ‘disagree’ (2), ‘agree’ (3), ‘agree strongly’ (4), and ‘not applicable’ (5). Raw scores range from 13 to 52. No score was calculated if no answer or ‘not applicable’ was chosen more than three times. Raw scores were converted to a theoretical score on a scale of 0 to 100. Higher scores indicate a higher level of patient activation. Patients were divided into four levels based on cut-off scores. In level 1 (score: ≤ 47.0), patients do not believe they have an important role regarding their health. In level 2 (score: 47.1–55.1), patients have lack confidence or knowledge to take action. In level 3 (score: 55.2–67.0), patients start to take action. In level 4 (score: ≥ 67.1), patients are maintaining active behavior [16]. The Dutch version of PAM-13 has been shown to be a reliable instrument [15]. Insignia Health provided a license.

### Predictor variables

Patient-, treatment- and health-related variables were measured though self-reported open and multiple-choice questions [see Additional file 1].

Basic patient-related characteristics included age, sex, highest degree of education, employment status, and living situation. Employment status was questioned as follows: “Do you currently work in paid employment”, with possible answers ‘no’, ‘part-time’, and ‘full-time’. In order to gain information on the level of activation in daily life, leisure-time activities, amount of home care services, presence or absence of children, and perception of social support, were measured. “Do you receive sufficient support from your environment?” with possible answers ‘yes’ or ‘no’, was asked to measure the perception of social support. Leisure-time activities were questioned as follows: “Do you have a hobby, do you do any sport, or are you a member of any organization?”

Treatment- and health-related factors included in this study were time since first dialysis, history of one or more renal transplantations, depressive symptoms, and self-reported health. Depressive symptoms were measured by the “Patient Health Questionnaire-2” (PHQ-2, Dutch version). This instrument is the short version of the PHQ-9 [17]. It questions frequency of a depressed mood and anhedonia (no interest in activities) during the last 2 weeks before the day of the study [17]. Answer possibilities were ‘Not at all’ (0), ‘Several days’ (1), ‘More than half the days’ (2), and ‘Nearly every day’ (3). The maximum score was 6. Our study used a cut-off score of 3, because sensitivity is 87% and specificity is 78% for major depression for this cut-off score [17]. Self-reported health was measured by the EuroQol Visual Analogue Scale (EQ-VAS, Dutch version) [18]. This 20-cm long visual analog scale is a part of the standardized EQ-5D. A scale of 0 to 100 was displayed. A score of 0 represented the worst health, and 100 the best health that could be imagined. A license from EuroQol Research Foundation was obtained.

### Statistical analysis

Analyses were performed using statistical package SPSS (version 23). Patient characteristics are presented as mean ± standard deviation for continuous variables and number and percentage for categorical variables.

Firstly, univariable linear regression procedures were conducted to examine associations between activation and all determinants. In advance dummy variables were created for all categorical determinants. Secondly, a multiple linear regression analysis with a stepwise exclusion method was conducted with all continuous and dummy variables. Determinants that seemed relevant for prediction of activation, were kept in the model (*p* <  0.05).

In order to detect existence and extent of multicollinearity in the final model, tolerance and variance inflation factor (VIF) were calculated [19]. By using histograms and scatter plots the assumptions of linearity, homoscedasticity and normality were checked and approved. Outliers were identified using Cook’s distance.

## Results

### Patient characteristics

**Fig. 1:**
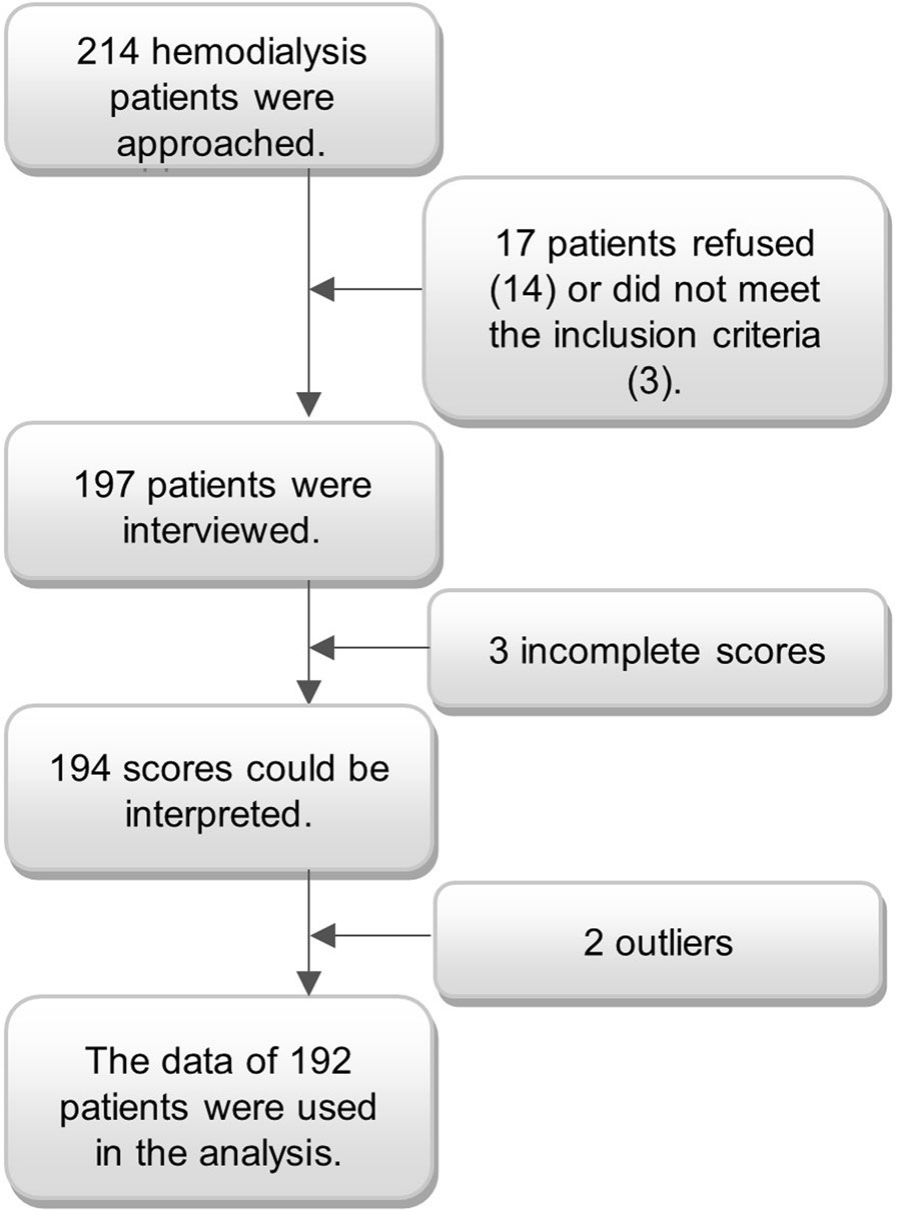
Flowchart of recruitment

A total of 214 patients undergoing hemodialysis were approached in this study of which 197 were willing and able to participate (response rate: 92%) (Fig. 1). Of these patients, 3 patients generated incomplete PAM scores and another 2 patients were excluded because of outlying results that affected the results of the analysis. Ultimately, data from 192 patients were used in the final analysis. Of these patients, 117 (61%) were male and age range was 20–95 years with a mean age of 72 ± 14 (Table 1). In these categories, the sample was representative for the population of patients undergoing dialysis in Flanders (Table 2). Patient characteristics and PAM scores are descripted in Table 1. A total of 138 (72%) participants completed the questionnaire together with the interviewer.

**Table 1:** Patient- and treatment-related characteristics and patient activation scores of the sample

Variable	Total *n*	*n* (%)	Mean, SD
Hospital	192		
Hospital 1 (university hospital)		92 (48)	
Hospital 2 (regional hospital)		46 (24)	
Hospital 3 (regional hospital)		54 (28)	
Sex, man	192	117 (61)	
Age, years	192		72 ± 14
Level of education	192		
Primary education		68 (35)	
Lower secondary education		29 (15)	
Higher secondary education		55 (29)	
Non-university higher education		32 (17)	
University education		8 (4)	
Employment status	192		
No work		175 (91)	
Full-time work		3 (2)	
Part-time work		14 (7)	
Perception social support	191		
Good social support		171 (90)	
Lacking social support		20 (10)	
Leisure-time activities	192		
Yes		83 (43)	
No		109 (57)	
Children	192		
Yes		149 (78)	
No		43 (22)	
Living condition	192		
Alone		49 (26)	
With someone		135 (70)	
Residential care home		8 (4)	
Care at home	192		
None		63 (33)	
1 service		47 (24)	
2 or more services		82 (43)	
Time since first dialysis	192		
3 months - 6 months		18 (9)	
> 6 months - 1 year		25 (13)	
> 1 year		149 (78)	
Renal transplantation	192		
Yes		17 (9)	
No		175 (91)	
EQ-VAS (0–100)	191		63 ± 17
PHQ-2	192		
No depressive symptoms (0–2)		168 (87.5)	
Depressive symptoms (3–6)		24 (12.5)	
Patient Activation Measure (0–100)	192		51 ± 10
Level 1		85 (44)	
Level 2		56 (29)	
Level 3		42 (22)	
Level 4		9 (5)

**Table 2:** Comparison sample and population Flanders

Age	Sample	Flanders [38]
Mean age (y)	72	72
Mean age men (y)	73	72
Mean age women (y)	70	73
Sex		
Male	61%	59%
Female	39%	41%
Legend: y = years		

### Identification variables associated with patient activation

Table 3 shows the results of univariable and multiple analysis with activation as a dependent variable associated with all independent variables.

**Table 3:** Univariable and multiple regression

		Univariable linear regression		Multiple linear regression R^2^: 0.306	
Variable	N	β	*p*-value	β	*p*-value
Age	192	−0.330	< 0.001*	− 0.284	< 0.001*
Self-reported health	191	0.328	< 0.001*	0.278	< 0.001*
Hospital	192				
Hospital 1					
Hospital 2		−0.165	0.033*	− 0.145	0.019*
Hospital 3		−0.016	0.833		
Sex, woman	192	−0.024	0.739		
Level of education	192				
Primary					
Lower secondary		0.083	0.285		
Higher secondary		0.088	0.275		
Non-university higher		0.219	0.005*		
University		0.212	0.004*		
Employment status	192				
No work					
Part-time		0.189	0.008*		
Full-time		0.152	0.033*		
Perception social support, good social support	191	−0.091	0.211		
Leisure-time activities, yes	192	0.331	< 0.001*	0.206	0.002*
Children, yes	192	−0.220	0.002*		
Living condition	192				
Alone		0.325	0.047*		
With someone		0.486	0.003*	0.141	0.025*
Residential care					
Care at home	192				
No services					
1 service		−0.010	0.900		
More than one service		−0.291	< 0.001*		
Time since first dialysis	192				
3 month – 6 month		−0.059	0.513		
> 6 month – 1 year					
> 1 year		−0.084	0.357		
Transplantation, yes	192	0.155	0.031*		
Depressive symptoms, yes	192	−0.135	0.063		

Legend: *: significant (*p* < 0.05)

β = standardized beta

#### Univariable linear regression

High activation scores correlate with lower age and high self-reported health. Patients in hospital 2 had significantly lower activation scores compared to patients in hospital 1. Higher activation scores were found in participants with a non-university higher degree or university degree compared to participants with only a primary education degree. Participants who worked full-time or part-time had higher activation scores, compared to participants who did not work. Having leisure-time activities and having children were related to patient activation. Patients who lived alone or with someone had a better level of activation compared to patients living in a residential care home. Patients who used more than one home care service had lower activation scores then patients who used no services. Participants with a history of kidney transplant had higher activation scores.

Activation score was not significantly related to sex, perception of social support, time since first dialysis, and depressive symptoms.

#### Adjusting for age

Age is a confounder for the relationship between activation and level of education, employment status, and renal transplant.

#### Multiple linear regression

The variance (R^2^) of the reduced multivariable linear model was 31%. When adjusted for all the other variables, age, self-reported health, hospital, leisure-time activities, and living situation were still associated with patient activation. Direction of the associations did not change, compared to univariable regression.

## Discussion

### Level of activation

One of the aims of this study was to investigate the level of activation of patients undergoing hemodialysis. The average activation score was 51 (± 10). Of the 192 patients, 44% patients did not believe they had an important role regarding their health. The high number of 73% of the patients did not take charge of their own health. The difficult combination of diet and fluid restrictions, strict medication regime, intensive dialysis treatment, comorbidities, and the proximity of healthcare workers three times a week, might explain the low activation scores in this population. Although the average activation score of 51 may be overestimated because people with cognitive impairment were excluded in the sample, activation of patients undergoing hemodialysis seems to be low and healthcare workers should be recommended to measure patient activation and intervene upon low levels of activation.

To further increase comprehensibility of the activation scores in our study, a literature search was performed about activation of other chronic patient groups which face similar challenges due to their disease. It appeared that patients with hypertension, depression, asthma, and diabetes have a higher average activation score compared to the patients undergoing hemodialysis in the present study [10, 20–30]. In the study of Bos-Touwen et al. a comparison was made between the average activation scores of patients with diabetes (55.3), chronic obstructive pulmonary disease (54.7), chronic heart failure (53.6), and chronic kidney disease (51.4) [10]. The kidney patients had the lowest activation score [10]. In the literature only one activation score was found to be lower than the average score measured in our study, namely the average activation score (50) of patients with osteoarthritis in South Korea [31]. Because of the various sample characteristics [22, 26], different countries of origin, cultural backgrounds, access to and cost of health care for patients [15, 22, 31], it can only be assumed and not determined with certainty that patients undergoing hemodialysis in Flanders have lower activation scores compared to other chronic patients.

### Multivariate analysis

The second aim was to identify patient- and treatment-related factors associated with activation. Age, self-reported health, hospital, leisure-time activities, and living situation were associated with activation in multiple analysis.

The R^2^ of the model was 0.306. Around 31% of variance of activation could be explained by these five variables. This R^2^ was higher than in previous models [10, 32].

It was demonstrated in our study and in many other studies that higher activation correlates with lower age [12, 15, 21, 22, 26, 30]. Explanations may be that older patients have lower self-efficacy [21], lower health literacy [33], seem to be less compliant [34], and are more accustomed to a paternalistic healthcare system [22] compared to younger people.

As in our study, previous literature showed that patients who reported being in good health, have higher patient activation [10, 15, 21, 26, 35]. This is not surprising, since patients who are more active, report better skills and better knowledge, confidence and behavior needed to manage their health condition [21, 26].

In our study, a significant lower level of activation was found in patients treated in hospital 2. This could be due to differences in the predialysis training, since previous research has shown that predialysis education can lead to higher levels of knowledge [36], and better self-management skills, such as better fluid balance [37]. However, we were unable to investigate this in our study, since no individual data about predialysis education was available. It would be interesting in future studies to investigate the association between organizational features of a hospital, accompaniment of patients before and during dialysis treatment, and patient activation.

When looking at functional disability and level of activation in daily life, previous research showed that patients who were more active, had no difficulties with activities of daily living [12], which could explain why active patients are more likely to have leisure time activities in our study. Although, Fowles et al. did not find a significant association between activation and membership of a health club [32]. Patients who live in a residential care home are less activated compared to patients living with someone. It was previously showed that people who live in their own house or apartment were significantly more activated than those living elsewhere [25].

In our study, age was a confounder in the association between patient activation and educational level, employment status, and history of renal transplant. The average 70-year-old population is less educated compared to a young population, more than half of the patients were on retirement, and older people with comorbidities might have lower chances to get on the transplant list.

### Strengths and limitations

To the best of our knowlegde, this study is the first study that has investigated patient activation specifically in the population of patients with hemodialysis.

Strengths of the study are a fairly large sample and a high response rate. Furthermore, the sample is representative for the Flemish hemodialysis population and participants were recruited in three hospitals. International validated questionnaires were used. Finally, because of the clinically meaningful outcome, the study provides information that is useful for practice.

Furthermore, the study has several limitations. First, no information about the directionality of the relationships could be obtained, due to the observational cross-sectional design. Second, data were measured using a self-reported questionnaire, which can induce recall bias or telescoping. However, only two questions had a recall timeframe. Third, certain possibly interesting variables were not included in this study, such as motivation, health literacy, hope, external control, cognitive impairment, genetics, life events, (the amount of) comorbidities, predialysis education on individual level, and organization features. Future research should take these variables into account. Fourth, in our sample, patients that were cognitively unable to understand difficult words were excluded from the study. In addition, no randomization techniques were used. These two factors might reduce the generalizability of our findings and might have created selection bias, which could have affected the results of the study.

### Recommendations

Nearly 73% of the patients did not take charge of their own health, which has shown to negatively influence health outcomes and costs. Hence, practitioners and other healthcare workers should be recommended to measure activation of patients and if needed to intervene upon low levels of patient activation.

Because older patients, who reported being in bad health, treated in a particular hospital, without leisure-time activities, and living in a residential care home, had significantly lower patient activation, patients at high risk can be identified using these screening factors. Furthermore, on the long term, provided that longitudinal studies show similar results and provide information on the directionality of the associations, these factors can help healthcare providers to develop personalized interventions to improve patient activation. Hence, improving self-reported health and/or encouraging patients to be more active in daily living could be part of these interventions.

Future research should investigate patient activation and its predictors in longitudinal research and provide information on the usefulness of these predictors in personalized interventions. Furthermore, the consequences of low activation should be further investigated in the particular population of patients undergoing hemodialysis.

## Conclusion

The average activation score of patients undergoing hemodialysis in Flanders was 51. Multiple linear regression revealed that age, self-reported health, hospital, leisure-time activities, and living situation explained 31% of the variance in activation. It seems that the average activation score of patients undergoing hemodialysis in Flanders is lower than the average activation score of patients with hypertension, asthma, depression, and diabetes.

Healthcare workers could be already recommended to measure the patient activation, and to take initiatives in order to increase it.

## Additional file

Additional file 1: Questionnaire used in the study. The survey questioned demographic, social and illness-related information. Moreover, the questionnaire on patient activation was also included. The participants have completed the Dutch translation of this questionnaire. (DOC 331 kb)

## Abbreviation

PAM: patient activation measure
