# Supplementary material for: Patient and treatment characteristics associated with patient activation in patients undergoing hemodialysis: a cross-sectional study
Source: BMC Nephrol. 2018 Jun 1;19:126. doi: 10.1186/s12882-018-0917-2 (PMC5984733; doi:10.1186/s12882-018-0917-2)
Supplement: Supplementary file 1 — Questionnaire used in the study. The survey questioned demographic, social and illness-related information. Moreover, the questionnaire on patient activation was also included. The participants have completed the Dutch translation of this questionnaire. (DOC 331 kb) [file 12882_2018_917_MOESM1_ESM.doc]

## Appendix 1: The questionnaire

## N.B. Participants completed the Dutch translation of this questionnaire.

Instructions for filling in the questionnaire:
• There are no right or wrong answers. It is important that we know your opinion.
• Read all questions carefully.
• Do not think too long, the first thought is usually the right one.
• Do not skip questions.
• Tick the box that suits you best and, if requested, complete the answer.
• If you would like to provide additional information, this may be noted alongside the questions.
• Filling in the questionnaire takes about 10 minutes.
• We want to know what you think yourself and not what the doctor or the researcher wants to hear.

Part 1: General information

1. What is your **date of birth**?

|  |  | / |  |  | / |  |  |  |  |
| --- | --- | --- | --- | --- | --- | --- | --- | --- | --- |

1. What is your **gender**?

- Man
- Woman

1. What is your highest **diploma**?

- Primary education
- Lower secondary education
- Higher secondary education
- Higher non-university education
- Higher university education
- Other:……………………………………………………………

Turn the page, please

1. Do you currently work in **paid employment**?
   - No
   - Yes
     - Yes, fulltime
     - Yes, parttime

Part 2: Social information

1. Do you receive sufficient **support from your environment**?
   - Yes
   - No
2. Do you have a **hobby**, do you do any **sport**, or are you a **member of any organization**?
   - No
   - Yes
     - Which? ………………………………………………………
3. What is your **family situation**?
   - Single
   - Having a partner
   - Widow/widower
4. Do you have **children**?
   - Yes
   - No
5. Which description suits your **living situation** best?
   - Living alone
   - Living together with partner
   - Living together with children
   - Living together with partner and children
   - Residential care home
   - Day center
   - Other: .....................................................................
6. Which **home care services** do you use?
   - Cleaning aid
   - Family help
   - Home nursing
   - Informal care (care from the immediate environment by family, neighbor, friend, ...)
   - No home care required
   - Other: ..................................................................

Part 3: Illness-related information

1. Which **dialysis treatment** do you receive?
   - Hemodialysis
   - Peritoneal dialysis
2. **How long** have you been treated with dialysis?
   - Between 3 month and 6 months
   - Between 6 months and 1 year
   - More than 1 year
3. Have you undergone a **kidney transplant**?
   - Yes
   - No

Turn the page, please

Best imaginable health state

1. On a scale from 0 to 100, how good or bad is
   **your health** today? Please write the number in
   the box below.

10

0

20

30

40

50

60

80

70

90

100

5

15

25

35

45

55

75

65

85

95

**YOUR HEALTH TODAY =**

Worst imaginable health state

*© 2010 EuroQol Group EQ-5D™ is a trade mark of the EuroQol Group*

5. Over the **past 2 weeks**, how often have you been bothered by any of the following problems? (Circle the answer that applies to you)

| Not at all | Several days | More than half the days | Nearly every day |
| --- | --- | --- | --- |

A. **Little interest or pleasure** in doing things

| Not at all | Several days | More than half the days | Nearly every day |
| --- | --- | --- | --- |

B. Feeling **down**, **depressed**, or **hopeless**
Below are some statements that people sometimes make when they talk about their health. Please indicate how much you agree or disagree with each statement as it applies to your personally by circling your answer. *There are no right or wrong answers, just what is true for you.*

Turn the page, please

Insignia Health. “Patient Activation Measure; Copyright © 2003-2010, University of Oregon. All Rights reserved.” Contact Insignia Health at www.insigniahealth.com

If the statement does not apply to you, circle N/A.

Blad omdraaien a.u.b.

| 1. | I am the person who is responsible for taking care of my health.   | Disagree strongly | Disagree | Agree | Agree strongly | N/A | | --- | --- | --- | --- | --- | |
| --- | --- | --- | --- | --- | --- | --- |
| 2. | Taking an active role in my own health care is the most important thing that affects my health.   | Disagree strongly | Disagree | Agree | Agree strongly | N/A | | --- | --- | --- | --- | --- | |
| 3. | I am confident I can help prevent or reduce problems associated with my health.   | Disagree strongly | Disagree | Agree | Agree strongly | N/A | | --- | --- | --- | --- | --- | |
| 4. | I know what each of my prescribed medications do.   | Disagree strongly | Disagree | Agree | Agree strongly | N/A | | --- | --- | --- | --- | --- | |
| 5. | I am confident that I can tell whether I need to go to the doctor or whether I can take care of a health problem myself.   | Disagree strongly | Disagree | Agree | Agree strongly | N/A | | --- | --- | --- | --- | --- | |
| 6. | I am confident that I can tell a doctor or nurse concerns I have even when he or she does not ask.   | Disagree strongly | Disagree | Agree | Agree strongly | N/A | | --- | --- | --- | --- | --- | |
| 7. | I am confident that I can carry out medical treatments I may need to do at home.   | Disagree strongly | Disagree | Agree | Agree strongly | N/A | | --- | --- | --- | --- | --- |   Insignia Health. “Patient Activation Measure; Copyright © 2003-2010, University of Oregon. All Rights reserved.” Contact Insignia Health at www.insigniahealth.com |
| 8. | I understand my health problems and what causes them.   | Disagree strongly | Disagree | Agree | Agree strongly | N/A | | --- | --- | --- | --- | --- | |
| 9. | I know what treatments are available for my health problems.   | Disagree strongly | Disagree | Agree | Agree strongly | N/A | | --- | --- | --- | --- | --- | |
| 10. | I have been able to maintain lifestyle changes, like healthy eating or exercising.   | Disagree strongly | Disagree | Agree | Agree strongly | N/A | | --- | --- | --- | --- | --- |   Insignia Health. “Patient Activation Measure; Copyright © 2003-2010, University of Oregon. All Rights reserved.” Contact Insignia Health at www.insigniahealth.com |
| 11. | I know how to prevent problems with my health.   | Disagree strongly | Disagree | Agree | Agree strongly | N/A | | --- | --- | --- | --- | --- | |
| 12. | I am confident I can work out solutions when new problems arise with my health.   | Disagree strongly | Disagree | Agree | Agree strongly | N/A | | --- | --- | --- | --- | --- | |
| 13. | I am confident that I can maintain lifestyle changes, like healthy eating and exercising, even during times of stress.   | Disagree strongly | Disagree | Agree | Agree strongly | N/A | | --- | --- | --- | --- | --- | |

## Thank you very much for completing the questionnaire!

Insignia Health. “Patient Activation Measure; Copyright © 2003-2010, University of Oregon. All Rights reserved.” Contact Insignia Health at www.insigniahealth.com
